# Identification and Characterization of Differentially Expressed MicroRNAs in Benign Prostatic Hyperplasia

**DOI:** 10.1002/cnr2.70178

**Published:** 2025-04-13

**Authors:** Lingmin Song, Xue Wang, Gang Wang, Liwei Zheng, Zhansong Zhou

**Affiliations:** ^1^ Department of Urologic Surgery Ningbo Yinzhou No. 2 Hospital Ningbo China; ^2^ Urological Research Institute of People's Liberation Army, Southwest Hospital, Army Medical University Chongqing China

**Keywords:** benign prostatic hyperplasia, microarray analysis, MicroRNA, qRT‐PCR

## Abstract

**Objectives:**

The primary aim of this research is to identify and describe the distinct patterns of microRNAs (miRNAs) that are unusually expressed in benign prostatic hyperplasia (BPH) tissues compared to normal prostatic tissues.

**Materials and Methods:**

The investigation began with the collection of three samples each from normal prostatic and BPH tissues. These samples underwent miRNA microarray analysis using the Agilent platform. Following the preliminary screening, a larger sample set, comprising five normal prostatic tissues and 36 BPH tissues, was subjected to qRT‐PCR to confirm the differential expression of the miRNAs initially identified.

**Results:**

The microarray analysis revealed that only miR‐126‐3p and miR‐4672 exhibited an expression profile marked by both a fold change > 1.5 and *p* < 0.05, indicating significant downregulation in BPH tissues. MiR‐145‐3p and miR‐143‐3p also showed downregulation with fold changes greater than 1.5; however, these changes did not reach statistical significance as their p‐values were above 0.05. Further attempts to validate these findings through qRT‐PCR did not confirm any notable dysregulation among the four miRNAs studied; the variations in their expression levels between normal and BPH tissues did not achieve statistical significance, with p‐values exceeding 0.1. From the data accrued, it can be inferred that the roles of miR‐4672, miR‐126‐3p, miR‐145‐3p, and miR‐143‐3p in BPH development continue to be an unresolved mystery, and the need for further investigation.

**Conclusions:**

This preliminary investigation establishes a foundation for subsequent studies aimed at elucidating the regulatory mechanisms underlying BPH. However, these results highlight the need for further investigation employing a more extensive sample size and comprehensive clinical data to elucidate their potential roles in the pathogenesis of BPH.

## 1 Introduction

Benign prostatic hyperplasia (BPH) is a common condition among middle‐aged and elderly adults, with its prevalence increasing with age. This disease is known to significantly impair the quality of life in affected individuals [1, 2]. A multitude of studies has investigated the pathogenesis of BPH, identifying key mechanisms such as androgen/estrogen imbalance, inflammatory processes, and the involvement of various growth factors [3]. However, the precise etiology of BPH remains elusive. An enhanced understanding of its pathogenesis could lead to improved treatment and prevention strategies for BPH.

MicroRNAs (miRNAs)—endogenous small non‐coding RNA strands typically spanning approximately 22 nucleotides‐are crucial for post‐transcriptional gene expression regulation [4]. MiRNAs play pivotal roles in various biological processes, such as development, cell proliferation, differentiation, and apoptosis [5]. Although traditionally, prostate cancer and BPH are considered distinct, the aberrant miRNA expression between these conditions suggests potential common molecular mechanisms. Some studies have revealed that altered miRNA expression serves as either a promoter or inhibitor of prostate cancer progression to regulate cellular activities, including proliferation, epithelial‐mesenchymal transition (EMT), metastasis, cellular metabolism, and the tumor microenvironment [6, 7, 8]. Similarly, in BPH, miR‐1202 and miR‐663AHG regulate cellular proliferation, apoptosis, and EMT in BPH by targeting specific pathways [9, 10]. Such regulatory capabilities highlight the potential of miRNAs as central players in prostate‐related diseases, including BPH.

Furthermore, research by Maristella Canovai et al. revealed that the exosomal secretion of miR‐210‐3p, miR‐183‐5p, and miR‐96‐5p affects drug resistance in prostate cancer cells [11]. However, emerging studies using rat models of BPH also have revealed the downregulation of miR‐128b, leading to increased EGFR expression and cell proliferation, changes that are preventable by canagliflozin, a potential therapeutic approach worth exploring in humans [12]. These two studies revealed similar mechanisms that may occur in prostate cancer and BPH and affect treatment responses. Additionally, wang et al. have proposed that miR‐29a/29b play a pivotal role in the development of BPH; these microRNAs are instrumental in the transformation of prostate stromal cells into myofibroblasts, a process that is induced by TGFβ1 [13]. However, it is well known that TGFβ1 is an important target in cancer therapy. Thus, there are some common links between miRNAs in regulating prostate cancer and BPH.

These miRNA alterations collectively suggest that, while research remains limited, miRNAs are not only biomarkers but also potential therapeutic targets in personalized management strategies for BPH, alongside their cancer implications.

In this study, we examined the miRNA expression profiles of BPH and normal prostate tissues utilizing miRNA microarray technology, followed by validation of select miRNAs through qRT‐PCR. Our study will contribute to the identification of novel microRNAs (miRNAs) involved in the pathogenesis of BPH, thereby offering theoretical underpinnings for the elucidation of miRNA‐regulated mechanisms in prostate hyperplasia. Additionally, it seeks to broaden the scope of research within this domain by providing new insights and directions.

## 2 Materials and Methods

### 2.1 Ethics Statement

This study is a retrospective case–control study. Normal prostate tissue and BPH tissue samples were obtained from Southwest Hospital, Army Medical University, No. 30, Gaotanyan Zhengjie, Shapingba District, Chongqing. The study was approved by the institutional review board at the participating site (protocol: 2015 Scientific Research No. 14), ensuring compliance with established ethical standards. The data are anonymous, and the requirement for informed consent was therefore waived [14].

### 2.2 Patients and Specimen Collection

In our study, normal prostate tissue and BPH tissue samples were unpaired. Five normal prostate tissue samples (aged 39 ± 8 years) were collected from donations post‐cardiac death. Each sample underwent rigorous histological evaluation via postoperative biopsies, which confirmed the absence of histological abnormalities, including prostatic intraepithelial neoplasia, prostate carcinoma, metastatic tumors, hyperplasia, or inflammatory conditions. Concurrently, a collection of 36 BPH tissue samples (aged 71 ± 7 years) was accrued during the six‐month period from January to June 2015. These samples were exclusively derived from patients undergoing transurethral resection of the prostate for BPH. Inclusion criteria mandated the exclusion of any individuals whose postoperative biopsies showed pathological findings, including prostate carcinoma, prostatic intraepithelial neoplasia, metastatic tumors, or histological signs of inflammation. Additionally, patients with urinary infections, diabetes mellitus, bacterial prostatitis, tumors in any organ, autoimmune diseases, or a history of recurrent BPH were also excluded from the study [15].

Immediately following surgical extraction, all fresh samples were cleansed with a cold, sterile 0.9% saline solution to remove contaminants. The transitional zone of each prostate was meticulously dissected, after which the samples were promptly frozen in liquid nitrogen and stored under these conditions until analysis [16].

### 2.3 MiRNA Microarray Analysis

Based on the inclusion criteria described above, we dispatched 14 tissue samples–comprising 7 samples each of BPH tissue and normal prostate tissue–to CapitalBio corporation (Captal‐Bio Corp. Beijing, China) in 2015. Initial sample quality assessment was performed by CapitalBio Corporation, focusing on total RNA integrity. Subsequently, they performed miRNA microarray analysis of three samples from each tissue type with intact total RNA according to the protocol cited in the literature in October 2015 [17]. Briefly described as follows: Total RNA extraction was carried out using Trizol (Invitrogen), followed by purification using the Kit (AM1561, Ambion, USA) according to the manufacturer's protocols [18]. The miRNA profiling utilized the Agilent miRNA array platform (Agilent Technologies Inc.), which includes probes for 2006 human mature miRNAs as cataloged in miRBase Release 21.0 and 2164 control probes from Agilent. To ensure the reliability of the data, each miRNA probe was replicated 30 times. Image capture, gridding, and data analysis were performed using Agilent's scanner software [19].

### 2.4 RNA Extraction and qRT‐PCR Analysis

The study conducted RNA extraction from an expanded sample set, comprising 36 BPH tissue samples and 5 normal prostate tissue samples. Total RNA was extracted using Trizol (Invitrogen), adhering rigorously to the manufacturer's guidelines. qRT‐PCR was conducted to measure miRNA expressions levels through Invitrogen's TaqMan probes within the CFX96 qPCR detection system (Bio‐Rad). Data normalization was facilitated by U6 snRNA, employing primers obtained from RiboBio (RiboBio Co. Ltd., Guangzhou, China). Quantitative analysis was performed using the 2ΔΔCT method [20].

### 2.5 Statistical Analysis

The miRNA array data were processed and analyzed using GeneSpring software V12 (Agilent Technologies Inc.), employing the default 90th percentile normalization method for data preprocessing. To select the differentially expressed genes, we used threshold values of ≥ 1.5 and ≤ −1.5‐fold change (fold change = *E*_experimental group_/*E*_control group_, *E*_experimental group_: the average expression level of the target miRNA under experimental conditions. *E*_control group_: the average expression level of the same miRNA under control conditions.) and a Benjamini‐Hochberg adjusted *p* value of 0.05. Data were Log^2^ transformed and median centered per gene via the Adjust Data function of CLUSTER 3.0 software, followed by hierarchical clustering using average linkage. Data analysis for the qPCR experiments was conducted using GraphPad Prism version 5.01 for Windows (GraphPad Software, California, USA), with group comparisons made via the independent t‐test. All tests were two‐tailed, with *p* < 0.05 deemed statistically significant [19].

## 3 Results

### 3.1 MiRNA Expression Profiling of BPH

**FIGURE 1:**
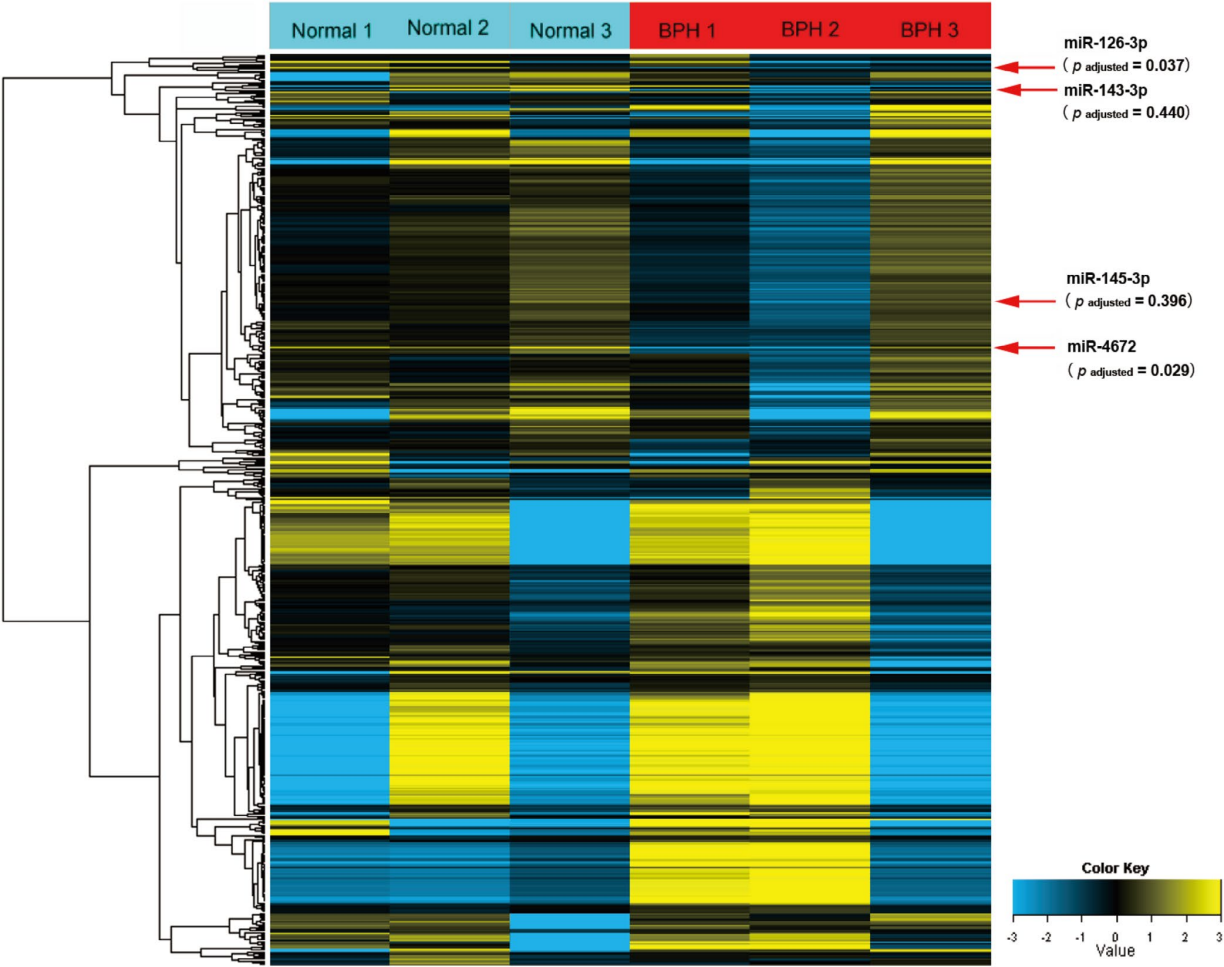
Heat map and hierarchical clustering of global miRNAs expression in normal prostate tissues and BPH tissues. Each row represents a miRNA, and each column represents a sample from either normal prostate tissues (normal 1, normal 2 and normal 3) or BPH tissues (BPH 1, BPH 2 and BPH 3). The color scale at the bottom denotes the relative expression levels of miRNA across all samples. BPH, Benign prostatic hyperplasia.

In this study, we analyzed miRNA expression profiles from three normal prostate and three BPH samples using miRNA microarray technology. The results suggested that miR‐126‐3p and miR‐4672 exhibited a fold > 1.5 and *p*_adjusted_ < 0.05, indicating significantly decreased levels in BPH tissues compared to normal prostate tissues (Figure 1 and Table 1). Although miR‐145‐3p and miR‐143‐3p were also down‐regulated in BPH tissues with a fold change exceeding 1.5, these changes did not reach statistical significance (*p*_adjusted_ > 0.05) (Table 1).

**TABLE 1:** The differential expression of miRNAs in BPH compared to normal prostate revealed by miRNA microarray data.

Systematic name	MiRNA microarray data			Chromosomal location
	Fold change	Regulation	*p*_adjusted_ value	
hsa‐miR‐126‐3p	2.35	Down	0.037	9:139565109–139 565 126[−]
hsa‐miR‐4672	1.68	Down	0.029	9:130631764–130 631 751[+]
hsa‐miR‐143‐3p	1.70	Down	0.440	5:148808547–148 808 561[−]
hsa‐miR‐145‐3p	1.67	Down	0.396	5:148810263–148 810 283[−]

Abbreviations: BPH, Benign prostatic hyperplasia; miRNAs, MicroRNAs.

### 3.2 qRT‐PCR Validation of Selected miRNAs

**FIGURE 2:**
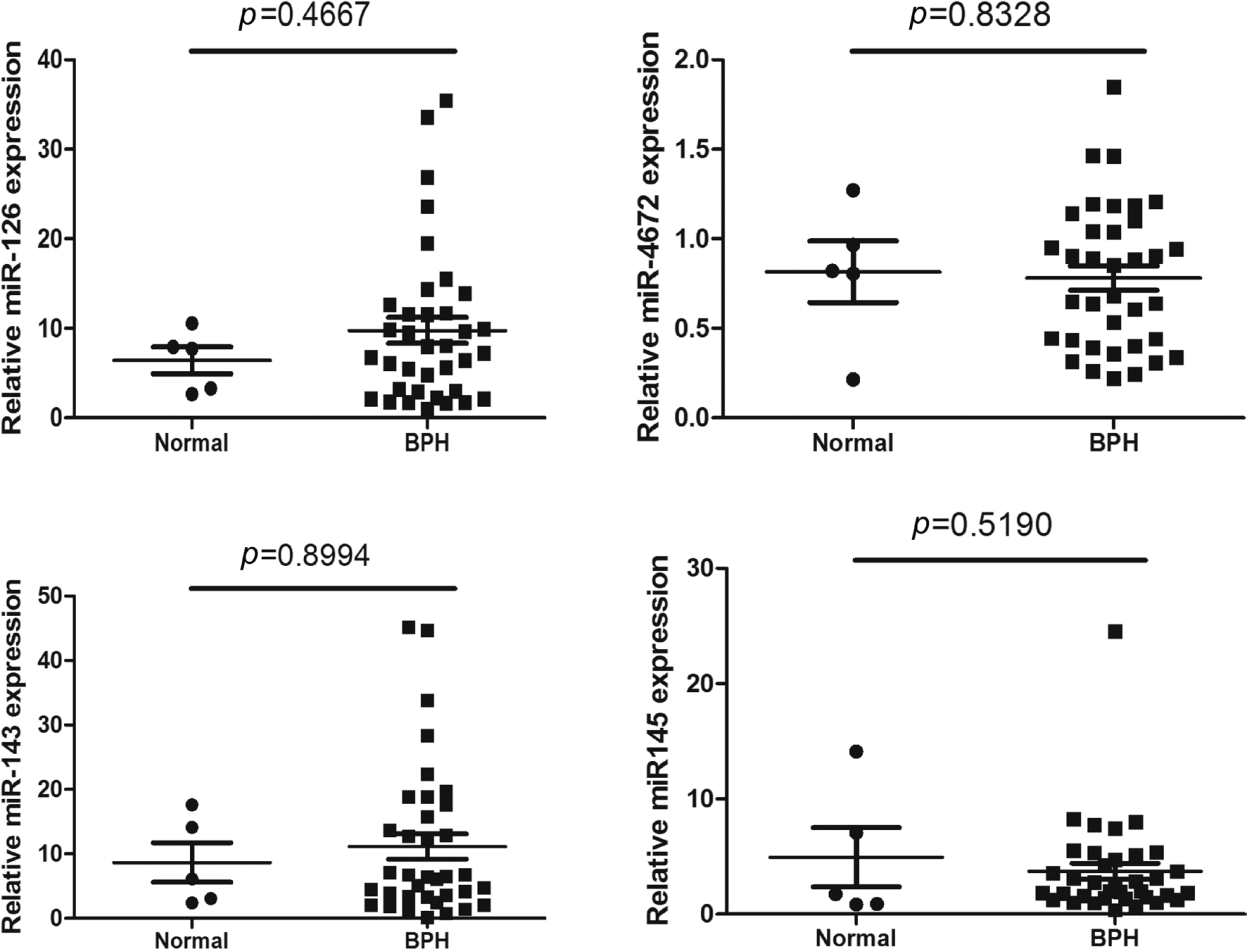
qRT‐PCR validation of microarray results. The relative expression levels of selected miRNAs were quantified using qRT‐PCR in 5 normal prostate tissues and 36 BPH tissues. *p*‐values were measured by an independent *t*‐test. BPH, Benign prostatic hyperplasia.

To validate the microarray findings, the sample size was increased and qRT‐PCR was conducted on 36 BPH samples and 5 normal prostate samples. The levels of miR‐145‐3p, miR‐143‐3p, miR‐126‐3p, and miR‐4672 exhibited minimal differences between the two groups, and these differences were not statistically significant (*p* > 0.1) (Figure 2).

## 4 Discussion

Although numerous studies have reported the mediating role of miRNAs in prostate cancer, few have investigated the regulatory role of miRNAs in BPH. To date, the role of miR‐4672 in either prostate cancer or BPH has not been reported. In our investigation, initial microarray analysis indicated a reduction of miR‐4672 expression in BPH; however, further evaluation using qRT‐PCR in an expanded sample set failed to demonstrate statistically significant differences. Although no other studies have explored the role of miR‐4672 in prostate cancer or BPH, some functions of this miRNA have still been identified. While the role of miR‐4672 in BPH or prostate cancer remains unexplored, existing literature provides insights into its functions in other contexts. The level of miR‐4672 is down‐regulated in exosomes from periodontal ligament cells, potentially influencing the migration of bone marrow mesenchymal stem cells by targeting IGF1 and KIT [21]. MiR‐4672 prevents hepatocyte steatosis and injury by directly targeting the 3′ UTR region of FABP1 [22]. The rs11275300:G allele reduces the affinity of hsa‐miR‐4672 for the 3′ UTR of DTL and is involved in poorer acral melanoma outcomes [23]. Additionally, a reduction of miR‐4672 levels in villous tissue is involved in recurrent spontaneous abortion [24]. It is evident that miR‐4672 is involved in important biological processes such as the migration of bone marrow mesenchymal stem cells, prognosis of melanoma, and recurrent miscarriage. However, its full range of functions remains to be further explored. Therefore, based on the low expression of miR‐4672 in BPH observed in our microarray analysis, we believe it is essential to investigate its role in BPH further.

We found that a decrease in miR‐126‐3p was observed in miRNA microarray analysis of BPH cases, yet subsequent verification using qRT‐PCR failed to confirm these findings. Extensive research on miR‐126‐3p found that it plays a crucial role in angiogenesis and inflammation, which are critical processes in the development of a range of human cancers and multiple diseases [25, 26]. Despite this, its effects on BPH have yet to be elucidated, and its involvement in prostate cancer remains insufficiently explored. A study found that miR‐126‐3p inhibits the Wnt/β‐catenin pathway, which is crucial for prostate development and proliferation of prostatic epithelial cells, by targeting LRP6 [27, 28]. Kyosuke Matsuzaki et al. proposed that miR‐126‐3p and miR‐30b‐3p in urothelial extracellular vesicles may be potential biomarkers for prostate cancer [29]. Recently, Liang Dong et al. highlighted the potential of miR‐126‐3p as a biomarker for forecasting lymph node invasion in high‐risk prostate cancer cases [30]. These findings suggest that reduced expression of miR‐126‐3p may contribute to BPH and prostate cancer. Further studies on miR‐126‐3p dysregulation are needed, which may help to gain insight into the pathogenesis of the disease.

In examining the potential role of miR‐145‐3p and miR‐143‐3p in BPH, analyses utilizing miRNA microarrays and qRT‐PCR with larger sample sizes did not find consistent aberrant expression of these miRNAs. This contrasts with findings by Nan Zhang et al., who reported a significant increase in hsa‐miR‐143‐3p in BPH patients compared to controls [2]. Such discrepancies might stem from inherent individual differences or variations in sample size and methodology. Exploring the functions of miR‐145‐3p and miR‐143‐3p in broader disease contexts offers insightful parallels that could enrich our understanding of their roles in BPH. For instance, C Coarfa et al.'s study identified significant suppression of these miRNAs in metastatic prostate cancer, where their re‐expression curtailed cell proliferation and impacted critical oncogenic pathways, including apoptosis, metastasis, cell cycle regulation, and Akt/mTOR signaling, as well as the androgen receptor axis [31]. Similarly, Dong Pan et al. highlighted that miR‐145‐3p or miR‐145‐5p‐mediated suppression of MTDH expression inhibited prostate cancer cell growth and metastasis [32]. Moreover, Yusuke Goto et al. identified that miR‐145‐3p significantly affected the survival outcomes of patients with castration‐resistant prostate cancer by targeting four critical molecules: MELK, NCAPG, BUB1, and CDK1 [33]. In a related context, miR‐143‐3p was shown to inhibit EMT in prostate cancer by targeting AKT1 [34] and to curb bone metastasis of Gleason 3+4 prostate cancer by targeting the LASP1‐mediated Wnt pathway [35]. Additionally, research on the co‐expression of miR‐145 and miR‐143 in colorectal cancers reveals that while these microRNAs are predominantly expressed in the colon, their expression is notably reduced in colorectal and other cancer types. Notably, the expression of these miRNAs is absent in colonic epithelial cells and instead significantly elevated in mesenchymal cells [36]. Therefore, the expression and function of miR‐145‐3p and miR‐143‐3p across various cancers and cellular contexts appear to be intricate and warrant deeper investigation. Given these diverse roles, understanding miR‐145‐3p and miR‐143‐3p in various cancers illuminates potential parallels and complexities worth investigating in BPH. Their differential expression and functional adaptability across tissue and disease contexts underscore the substantial need for deeper exploration to uncover potential therapeutic targets or diagnostic markers relevant to BPH.

However, there are several limitations in this study that should be considered. Firstly, the sample size was relatively small, particularly with respect to normal prostate tissue, which poses challenges in ensuring statistical power and generalizability of the findings. The limited number of samples, combined with potential individual variability in both patient characteristics and tissue samples, may introduce bias into the results, affecting the reliability of statistical analyses. Additionally, the difficulty in obtaining sufficient normal prostate tissue from human patients for comparison further compounds this issue. To address this limitation, future studies could consider utilizing animal models that closely resemble human prostate tissue. These models would provide a more robust means of exploring the biological relevance of miR in prostate health and disease, and help overcome the challenges related to tissue availability and sample size.

## 5 Conclusion

Microarray analysis identified reduced expression of miR‐126‐3p and miR‐4672 in BPH tissues. However, qPCR results did not verify that the differential expression levels of miR‐126‐3p, miR‐4672, miR‐143‐3p, and miR‐145‐3p in BPH were statistically significant. Therefore, the specific mechanism of the involvement of these miRNAs in BPH remains to be determined. Despite this, the study provides a foundational basis and stimulates potential research ideas for further exploration of the role of miRNAs in BPH.

## Author Contributions

The study's conceptualization and design were significantly shaped by Z.Z. and L.S. has drafted the manuscript and made acquisition and analysis of data. Z.Z. has provided the final endorsement of the version slated for publication. X.W. has engaged in the manuscript's review and editorial processes. G.W. and L.Z. helped in drafting and visualizing the manuscript. All participating authors have read and ratified the manuscript in its final form.

## Ethics Statement

Human tissue samples were obtained from Southwest Hospital, Army Medical University, following approval from the Ethics Committee. All methodological enactments involving human subjects were conducted following the ethical standards of the institutional and national research committees, in accordance with the 1964 Helsinki declaration and its later amendments, or comparable ethical standards.

## Conflicts of Interest

The authors declare no conflicts of interest.

## Data Availability

All data created or examined during this research are included in this published paper. The corresponding author may allow access to the used or analyzed datasets in this study upon a reasonable request.
